# Generation of a Useful *roX1* Allele by Targeted Gene Conversion

**DOI:** 10.1534/g3.113.008508

**Published:** 2013-11-26

**Authors:** Manasi S. Apte, Victoria A. Moran, Debashish U. Menon, Barbara P. Rattner, Kathryn Hughes Barry, Rachel M. Zunder, Richard Kelley, Victoria H. Meller

**Affiliations:** * Department of Biological Sciences, Wayne State University, Detroit, Michigan 48202; † Department of Biology, Tufts University, Medford, Massachusetts 02155; ‡ Department of Molecular and Human Genetics, Baylor College of Medicine, Houston, Texas 77030

**Keywords:** *roX*, dosage compensation, gene conversion, mutagenesis

## Abstract

Methods for altering the sequence of endogenous *Drosophila melanogaster* genes remain labor-intensive. We have tested a relatively simple strategy that enables the introduction of engineered mutations in the vicinity of existing *P*-elements. This method was used to generate useful alleles of the *roX1* gene, which produces a noncoding RNA involved in dosage compensation. The desired change was first introduced into a genomic clone of *roX1* and transgenic flies were generated that carry this sequence in a *P*-element. Targeted transposition was then used to move the *P*-element into *roX1*. Remobilization of the targeted insertion produced large numbers of offspring carrying chromosomes that had precisely introduced the engineered sequences into *roX1*. We postulate that this occurred by gap repair, using the *P*-element on the sister chromatid as template. This strategy was used to introduce six MS2 loops into the *roX1* gene (*roX1^MS2-6^*), enabling detection of *roX1* RNA by a MCP-GFP fusion protein in embryos. The *roX1^MS2-6^* remains under the control of the authentic promoter and within the correct genomic context, features expected to contribute to normal *roX1* function. The ability to replace relatively large blocks of sequence suggests that this method will be of general use.

The *roX1* and *roX2 (RNA on the X-1* and *-2*) are noncoding transcripts that play a central role in sex chromosome dosage compensation in flies. This process ensures a constant ratio of X-linked to autosomal gene products in males that have a single X chromosome. A complex of proteins and *roX* RNA [the Male Specific Lethal (MSL) complex] is recruited to X-linked genes. This complex directs chromatin modifications that result in increased expression from X-linked genes (Smith *et al.* 2001; Deng and Meller 2006; Conrad and Akhtar 2011; Larschan *et al.* 2012). The *roX* RNAs are essential for X localization of the intact complex and, despite their lack of sequence similarity, are functionally redundant (Meller and Rattner 2002). Expression of *roX* RNA from an autosomal transgene will rescue *roX1 roX2* males. However, both *roX* genes are X-linked, and both can recruit the MSL complex to chromatin adjacent to sites of *roX* transcription (Kelley *et al.* 1999; Kageyama *et al.* 2001; Park *et al.* 2003; Oh *et al.* 2004). This suggests that the function of the *roX* genes depends, in part, on their situation on the X chromosome.

During *P*-element–induced mutagenesis of *roX1*, we observed numerous identical rearrangements. These appear to be produced by a highly favored gene conversion that replaces more than 1 kb of *roX1* with sequence contained within a *P*-element inserted in *roX1*. Replacement is driven by homology between genomic sequence flanking the insertion site and within the *P*-element. We tested this as a general strategy for gene engineering by introducing RNA loops from the MS2 virus (MS2 loops) into the endogenous *roX1* gene, creating *roX1^MS2-6^*. RNAs that contain MS2 loops can be visualized *in vivo* when a fusion of GFP to the MS2 loop-binding protein (MCP-GFP) is expressed (Bertrand *et al.* 1998). The *roX1^MS2-6^* allele preserves the normal chromatin context of *roX1* and lacks all *P*-element sequence. Activity of *roX1^MS2-6^* in dosage compensation is indistinguishable from that of wild-type *roX1*. We have named the replacement strategy “targeted gene conversion” (TGC). TGC is technically simple and capable of introducing large blocks of nonhomologous sequence. It is able to replace sequences that are more than 1 kb from a *P*-element insertion. The strategy that we tested relies on a *P*-element near the site to be mutated. However, recently developed methods for directed mutagenesis may enable a modified form of TGC in regions that lack *P*-elements.

## Materials and Methods

### Fly culture

Flies were raised on a yeast, molasses, and cornmeal diet at room temperature. Mutations are described in citations or Lindsley and Zimm (1992).

### Gene conversion using an autosomal template

The p[*w^+mC^* GM *roX1^MS2-6/12^*] transgenes were generated by inserting 6 or 12 MS2 loops into a *Bgl*II site in a 4.9-kb genomic *Eco*R1 fragment containing *roX1*. Males with autosomal insertions of these transgenes were mated to *w roX1^Δ891^ Df(1)52/ Binsincy* virgins to generate *w roX1^Δ891^ Df(1)52/* Dp(1;Y) *B^s^ v^+^ y^+^*; p[*w^+mC^* GM *roX1^MS2-6/12^*]/+ males. *Df(1)52* removes *roX2* and nearby essential genes. Males are rescued by a duplication of the *roX2* region on the Y chromosome. These males were mated to *C(1)DX y^1^ f^1^/* Dp(1;Y) *B^s^ v^+^ y^+^*; p[*ry^+^*Δ2-3]99/+ females to produce *w roX1^Δ891^ Df(1)52*; p[*w^+mC^* GM *roX1^MS2-6/12^*]/ p[*ry^+^*Δ2-3]99 dysgenic sons that were mated to *C(1)Dx y^1^ f^1^*; p[4Δ4.3] females. The cosmid p[4Δ4.3] restores all essential genes removed by *Df(2)52*, but it is deleted for *roX2* and *w^+mC^* (Meller and Rattner 2002). If the break created by mobilization of *roX1^Δ891^* was repaired by copying *roX1* sequence within p[*w^+mC^* GM *roX1^MS2-6/12^*], then this would result in loss of the *w^+^* marker, restoration of *roX1* activity, and incorporation of MS2 loops into *roX1*. White-eyed sons were mated individually to *C(1)Dx y^1^ f^1^*; p[4Δ4.3] females and MS2 loop incorporation was determined by PCR of single fly squashes.

### Targeted transposition

The p[*w^+mC^* GM *roX1^MS2-6^*] transgene was moved into *roX1* by targeted transposition, using the *roX1^mb710^* plArB element as the target site. Dysgenic males (*y w roX1^mb710^*; p[*w^+mC^* GM *roX1^MS2-6^*] / *Sb* p[*ry^+^*Δ2-3]99B/+) were mated to *C(1)Dx y^1^ f^1^* females. Hops (*w^+mC^ Sb* sons) were collected and individually mated to *C(1)Dx y^1^ f^1^* females. X-linked insertions were mapped by *in situ* hybridization. Insertions close to *roX1* (3F) were characterized by single fly PCR to verify the presence and orientation, of p[*w^+mC^* GM *roX1^MS2-6^*]. Outward-facing primers [plac1(+), pry4(+), and pry2] (Supporting Information, Table S1) in P-ends were paired with each other or with primers in *roX1* (BPR10, BPR15) to determine the arrangement of tandem insertions. Primers are presented in Table S1. Targeted transpositions are designated as *roX1^[MS2-6]TXX^* (tandem insertion) or *roX1^[MS2-6]RXX^* (replacement of plArB), followed by the transposition number.

### Gene conversion in males

Three independent targeted transpositions of p[*w^+mC^* GM *roX1^MS2-6^*] in *roX1* were remobilized with p[*ry^+^*Δ2-3]99. Lines *roX1^[MS2-6]T2A^* and *roX1^[MS2-6]T4B^* retain plArB in tandem, and *roX1^[MS2-6]R36A^* has replaced plArB with p[*w^+mC^* GM *roX1^MS2-6^*]. Dysgenic males were mated to *C(1)Dx y^1^ f^1^* females. White-eyed sons were individually mated to *C(1)Dx y^1^ f^1^* females. Introduction of MS2 loops and retention of *P*-element sequences was determined by PCR. The *roX1* primers flanking the MS2 loops (*roX1*^ex6^F and BPR19) amplify 547 bp from *roX1^+^* and 869 bp when MS2 loops are inserted (*roX1^MS2-6^*).

### Gene conversion in females

The targeted transposition *roX1^[MS2-6]T2A^* was mobilized in females. A total of 244 dysgenic females (*roX1^[MS2-6]T2A^* / Binsincy; *Sb* p[*ry^+^*Δ2-3]99/+) were mated to *yw* males, with approximately 10 females per vial; 25 out of 26 vials produced at least one white-eyed nonbalancer son, indicating excision. Two hundred sixty-nine excisions were mated individually to *C(1)Dx y^1^ f^1^* females. A randomly selected subset of these was analyzed by PCR for MS2 loop incorporation and loss of *P*-element sequences.

### DNA blotting

DNA from 100 flies was extracted as described (http://www.fruitfly.org/about/methods/inverse.pcr.html). DNA was suspended in 300 μl DEPC water and treated with RNAse A and proteinase K; 15 μg DNA was digested overnight with *Eco*RI, concentrated, electrophoresed, and transferred to a charged nylon membrane. Blots were probed with a ^32^P-labeled, 2.03-kb *Eco*R1-*Mlu*1 fragment spanning the promoter and 5′ end of *roX1* using previously described methods (Church and Gilbert 1984). Restriction digests of a 4.9-kb *roX1* genomic clone served as a molecular weight marker.

### Visualization, photography, and image processing

Immunodetection of MSL1 on polytene preparations was performed as previously described (Kelley *et al.* 1999). MCP-GFP is removed by acetic acid fixation, preventing visualization on polytene chromosomes. To visualize MCP-GFP recruitment in embryo nuclei, homozygous *roX1^MS2-6^ roX2Δ*; [*w^+mC^* MCP-GFP] females were mated to males carrying a p[*w^+mC^ sqh-mCherry*] insertion on the X chromosome. Male embryos are distinguished by lack of *mCherry* signal. The 3-h to 12-h embryo collections were dechorionated, fixed in 4% paraformaldehyde with 0.1% Tween-20, DAPI-stained, and mounted with DABCO anti-fade agent in 50% glycerol. Z-stacks were recorded for individual embryos using an Olympus Fluoview FV10i scanning confocal microscope with a 60× water/oil immersion lens. Images were processed by converting to 8-bit format and importing individual Z-stacks into ImageJ. Because *mCherry* signal was weak and diffuse, the brightness of this channel was uniformly enhanced for reproduction (Figure 4, C, H, and M). Consistent patterns of GFP localization were observed in images of more than 30 embryos from three collections.

## Results

### An autosomal *roX1^MS2-6^* transgene restores X chromosomal MSL1 localization

**Figure 1:**
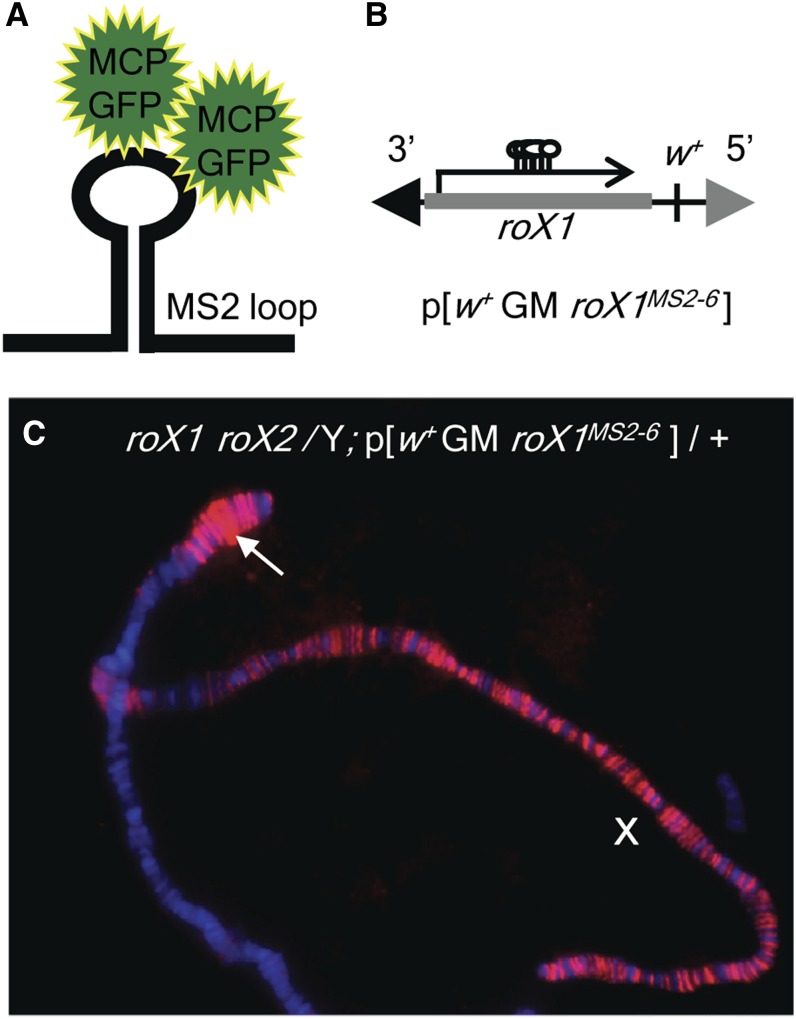
The *roX1^MS2-6^* restores X-chromosome MSL localization. (A) MS2 loops in RNA enable transcript visualization with MS2 coat protein (MCP) fused to GFP. (B) Structure of the p[*w^+mC^* GM *roX1^MS2-6^*] transgene. Six tandem MS2 loops (322 bp) are inserted in a 4.9-kb genomic *roX1* clone. (C) Polytene chromosomes from a male *roX1^ex6^roX2Δ /Y*; p[*w^+mC^* GM *roX1^MS2-6^*] /+ larva were immunostained with MSL1 antibody detected by Texas Red. DNA is counterstained with DAPI. Restoration of X localization and spreading of MSL1 into the autosome flanking the p[*w^+mC^* GM *roX1^MS2-6^*] insertion site (arrow) is observed.

RNA accumulation can be visualized in tissues or chromosome preparations by *in situ* hybridization. Although useful, this method is time-consuming and incompatible with living tissue. RNAs that contain stem loops from the MS2 virus can be visualized *in vivo* when a fusion of MCP-GFP is expressed (Figure 1A) (Bertrand *et al.* 1998). A *roX1* transgene was constructed with six MS2 loops (*roX1^MS2-6^*) inserted in a region previously shown to be nonessential (Figure 1B) (Stuckenholz *et al.* 2003; Deng *et al.* 2005). An autosomal copy of this transgene, p[*w^+mC^* GM *roX1^MS2-6^*], rescues X-localization of a key member of the MSL complex, Male-Specific Lethal 1 (MSL1), in *roX1 roX2Δ* males (Figure 1C). However, ectopic recruitment surrounding the site of transgene insertion is also observed (arrow, Figure 1C). Fully wild-type behavior of *roX1* is consequently expected to require expression from the X chromosome, possibly from the *roX1* locus itself.

### Gene conversion by repair using a sister chromatid template

During *P*-element mutagenesis of *roX1*, we obtained a series of mutations that suggested a strategy for inducing precise changes in target genes. A reporter construct containing the *roX1* promoter fused to LacZ (p[*w^+mC^ roX1P*-βgal]) was moved into *roX1* in an effort to capture enhancers in the vicinity. This was accomplished by targeted transposition to the plArB element in *roX1^mb710^* (Figure S1 A, B) (Gloor *et al.* 1991; Heslip and Hodgetts 1994). The resulting insertion, *roX1^w+tandem^*, retained plArB and is marked with *w^+mC^*, facilitating subsequent mutagenesis. Hybrid element insertion was used to generate *roX1^Δ891^*, deleted for the plArB element and 891 bp flanking the insertion site, but retained p[*w^+mC^ roX1P*-βgal] (Preston and Engels 1996; Preston *et al.* 1996) (Figure S1C). Remobilization of *roX1^Δ891^* produced numerous white-eyed offspring from virtually every dysgenic parent. However, only a few imprecise excisions were identified (Figure S2A). Instead, almost 70% of excisions carried molecularly identical rearrangements exemplified by the severe *roX1^SMC17A^* allele (Figure S1D). These appear to be produced by a gene conversion that occurs when the double-stranded break produced by *P*-element mobilization undergoes gap repair using a sister chromatid template (Figure S2B). The rearrangement generated is consistent with repair driven by homology between the *roX1* promoter on the broken chromosome more than 1 kb from the break site and in p[*w^+mC^ roX1P*-βgal]. Homology is also shared by terminal inverted repeats at the 5′ and 3′ *P*-element ends (P-ends). In all 38 flies recovered with this rearrangement, the 3′ P-end has been precisely replaced by the 5′ P-end, a structure consistent with the proposed mechanism of repair. These chromosomes lost 1.2 kb of *roX1* sequence flanking the p[*w^+mC^ roX1P*-βgal] insertion site and replaced it with more than 3 kb of LacZ sequence fused to the *roX1* promoter. This mechanism is thus capable of efficiently replacing large regions close to *P*-elements.

### Lack of repair utilizing a template on a different chromosome

To determine if efficient gene conversion was an intrinsic property of the *roX1* locus that is independent of template location, we attempted to generate a useful allele of *roX1* by introducing sequence from an engineered *roX1^MS2-6^* transgene situated on an autosome. Gene conversion at *white* (*w*) occurs in a small percentage of excisions when a *P*-element is mobilized from *w* and a template with homology to insertion site is present in the genome (Banga and Boyd 1992; Johnson-Schlitz and Engels 1993; Nassif *et al.* 1994; Lankenau *et al.* 1996). We attempted to introduce MS2 loops into *roX1* from an autosomal p[*w^+mC^* GM *roX1^MS2-6^*] template. Dysgenic males with a p[*w^+mC^* GM *roX1^MS2-6^*] donor on the third chromosome and the *roX1^Δ891^* target site on the X chromosome were generated. To enable phenotypic detection of gene conversion, the target X chromosome was also deleted for *roX2* (see *Materials and Methods* for full description of genotypes and matings). The *roX1^Δ891^* is a severe loss-of-function mutant. Conversion to *roX1^MS2-6^* will restore male viability and eliminate the *w^+mC^* marker in *roX1^Δ891^*. Approximately 100 white-eyed sons were recovered and tested by PCR for incorporation of MS2 loops, but only wild-type *roX1* sequences were detected. Although a gene conversion strategy utilizing a template situated on another chromosome may be productive in some situations, it was not useful in this instance.

### Targeted transposition of p[*w^+mC^* GM *roX1^MS2-6^*]

**Figure 2:**
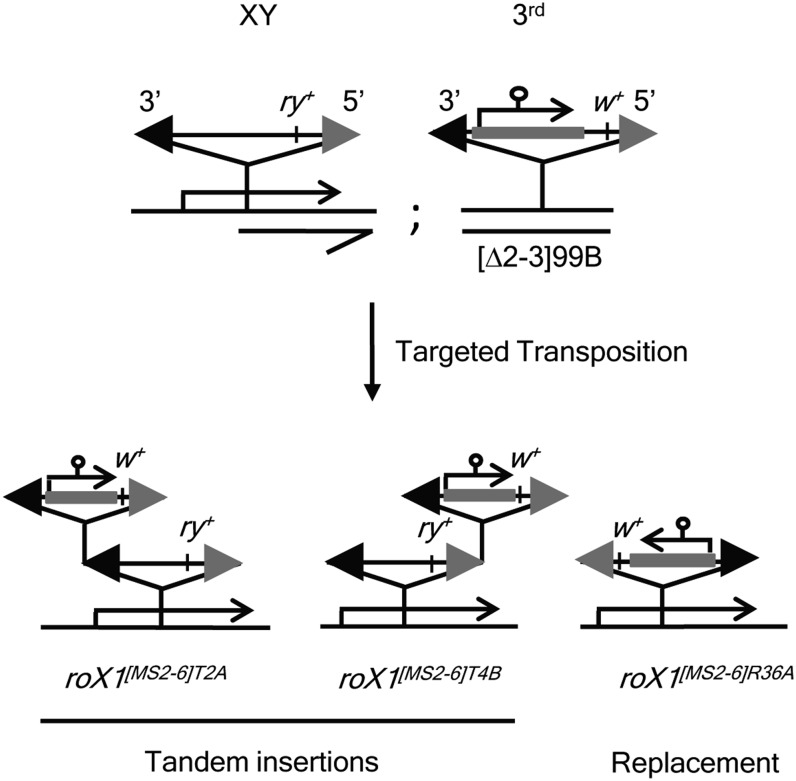
Strategy for targeted transposition into *roX1*. (Top) A p[*w^+mC^* GM *roX1^MS2-6^*] insertion on the third chromosome was mobilized in *roX1^mb710^* males with plArB (*ry^+^*) in *roX1*. (Bottom) Tandem insertions (*roX1^[MS2-6]T2A^*^,^ *roX1^[MS2-6]T4B^*) retain plArB. The *roX1^[MS2-6]R36A^* is a precise replacement of plArB by p[*w^+mC^* GM *roX1^MS2-6^*].

To determine if p[*w^+mC^* GM *roX1^MS2-6^*] would be utilized for gap repair if situated in *roX1*, targeted transposition was used to move it to the plArB insertion site in *roX1^mb710^* (Figure 2). Mobilization produced abundant hops to the X chromosome, 68% of which (34/50 insertions) were in *roX1*. The reason for the unusually high efficiency of targeting is unknown, but an interaction of *roX* genes in the male germ line, where transposition occurred, is suggested. Insertions on the X chromosome were characterized by *in situ* hybridization and PCR. The plArB was retained in tandem with 32 of the insertions. However, two precise replacements of plArB with p[*w^+mC^* GM *roX1^MS2-6^*] were recovered.

### Mobilization of targeted insertions to create *roX1^MS2-6^*

**Figure 3:**
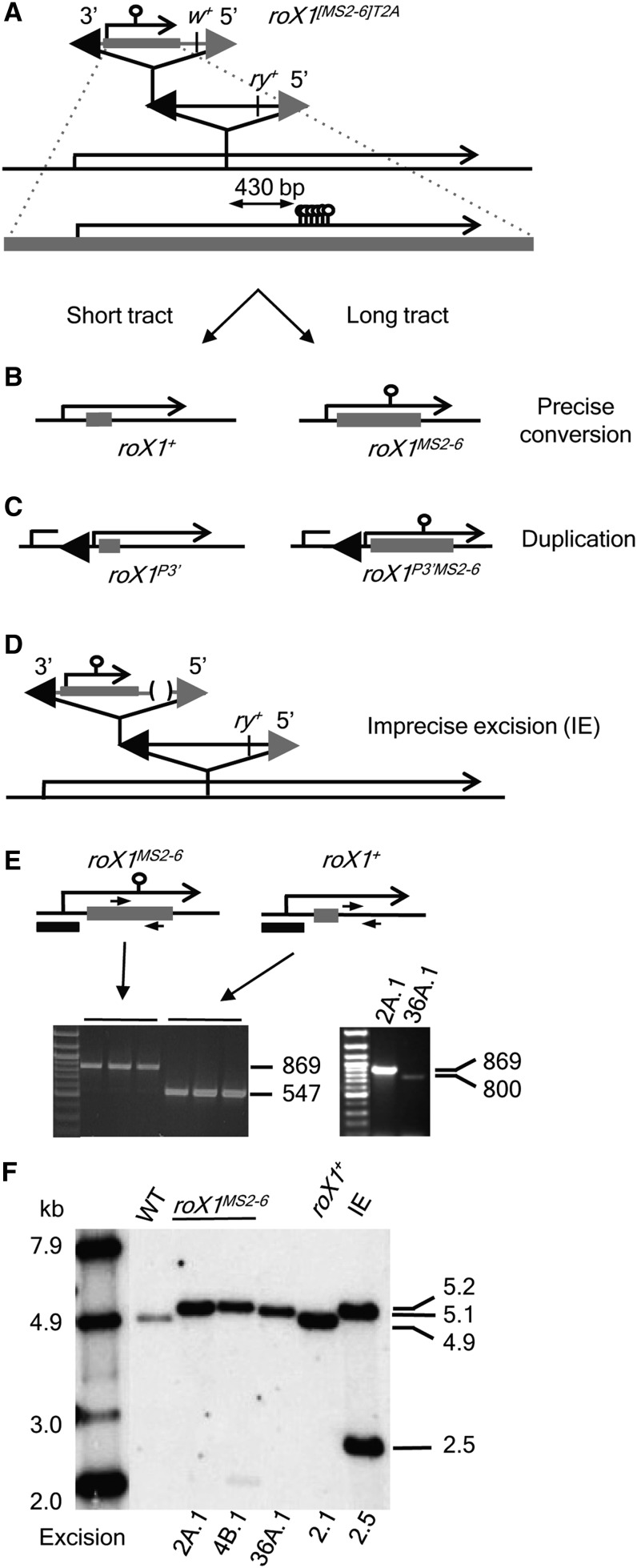
All predicted products of homology-dependent gene conversion are recovered. (A) The *roX1^[MS2-6]T2A^* is a tandem insertion of p[*w^+mC^* GM *roX1^MS2-6^*] at the 3′ end of plArB. Alignment of the engineered *roX1^MS2-6^* (gray line) is shown collinear to and below the corresponding genomic sequence. The MS2 loops are 430 bp from the plArB insertion site. (B and C) Predicted products of homology-dependent gap repair and gene conversion. Left panels depict short repair tracts that do not incorporate MS2 loops; right panels depict longer tracts incorporating MS2 loops into the repaired chromosome. (B) Homology in *roX1* precisely substitutes a portion of *roX1^[MS2-6]T2A^* (thick gray line) at the plArB insertion site. (C) Homology in *roX1* and at P-ends leads to retention of the 3′ P-end and duplication of 5′ *roX1* sequence. (D) An imprecise excision removing *w*^+mC^ from *roX1^[MS2-6]T2A^*. (E) MS2 loop incorporation was detected by PCR using primers (arrows) flanking the MS2 loop insertion site (top). The *roX1^MS2-6^* produces an 869-bp amplicon and *roX1^+^* produces a 547-bp amplicon. Three representative excisions in each category are shown. Contraction of the MS2 loop array in excision 36A.1 was detected by a reduction of the amplicon to 800 bp (right). (F) Blot of *Eco*R1-digested DNA probed with the *roX1* promoter (black bar, E). Hybridization to a single 4.9-kb *roX1* fragment is seen in wild-type (WT) flies and in a gene conversion that did not incorporate MS2 loops or retain a P-end (*roX1^+^*). A single 5.2-kb fragment is detected in two precise conversions incorporating MS2 loops (lines 2A.1 and 4B.1). Hybridization to a single 5.1-kb band is observed in excision 36A.1, consistent with the reduced MS2 loop array observed by PCR. Line 2.5 is the imprecise excision depicted in (D). A 5.2-kb band from p[*w^+mC^* GM *roX1^MS2-6^*] and a 2.5-kb band produced by disruption of genomic *roX1* by insertion of plArB are present.

Three targeted insertions in *roX1* were remobilized: a replacement line (*roX1^[MS2-6]R36A^*) and two tandem insertions with different orientations (*roX1^[MS2-6]T2A^* and *roX1^[MS2-6]T4B^*) (Figure 2). Dysgenic males (*roX1^[MS2-6]XX^*; *Sb* p[*ry^+^*Δ2-3]99/ +) were mated to C(1)DX *y^1^ f^1^* females. Mobilization is very frequent, with more than 90% of dysgenic males producing white-eyed sons, which comprise ∼20% of male offspring. White-eyed sons were mated individually to C(1)DX *y^1^ f^1^* females and analyzed by PCR for repair of *roX1* and inclusion of MS2 loops. Amplicons spanning the MS2 loop insertion site produce products characteristic of both wild-type *roX1* (547 bp) and *roX1^MS2-6^* (869 bp) from targeted transpositions, but almost 99% of white-eyed offspring produced a single amplicon. A total of 352 excisions were analyzed (169 for *roX1^[MS2-6]T2A^*, 103 for *roX1^[MS2-6]T4B^*, 80 for *roX1^[MS2-6]R36A^*). Regardless of the starting line, more than 10% of white-eyed sons had incorporated MS2 loops into the repaired chromosome (Figure 3, B–E and Table 1). Amplicons from representative flies containing MS2 loops were sequenced, confirming faithful copying. The MS2 loops are 322 bp of nonhomologous sequence situated 430 bp from the point of *P*-element insertion (Figure 3A). Incorporation of MS2 loops therefore requires a gene conversion tract more than 750 bp in length. However, three flies generated by mobilization of *roX1^[MS2-6]R36A^* produced 800 bp PCR amplicons, consistent with contraction of the MS2 loop array during gene conversion (Figure 3E).

**Table 1:** Rearrangements recovered during generation of *roX1^MS2-6^*

Targeted Transposition	Excisions Analyzed	No MS2 Loop Incorporation	MS2 Loop Incorporation	Imprecise Excisions
*roX1^[MS2-6]T2A^*	169	150 (88.75%)	18 (10.65%)	2 (1.18%)
*roX1^[MS2-6]T4B^*	103	90 (87.37%)	12 (11.65%)	1 (0.97%)
*roX1^[MS2-6]R36A^*	80	71 (88.75%)	8 (10%)	1 (1.25%)

Our aim was to engineer *roX1* without leaving vector or *P*-element sequence behind. However, homology at P-ends can support gap repair, leading to predictable rearrangements. When the tandem insertion *roX1^[MS2-6]T2A^* is mobilized, homology-dependent gap repair can restore *roX1* with no *P*-element sequences or with a 3′ P-end retained (Figure 3, B and C). Flies that retain the 3′ P-end also duplicate the 5′ end of *roX1* and, depending on the length of repair tract, have full-length wild-type *roX1* (*roX1^P3′^*) or *roX1* with MS2 loops (*roX1^P3′MS2-6^*) (Figure 3C). Retention of the 3′ P-end is also possible following mobilization of the replacement line *roX1^[MS2-6]R36A^* (Figure S5). When the tandem insertion *roX1^[MS2-6]T4B^* is mobilized, the 3′ end of p[*w^+mC^* GM *roX1^MS2-6^*] as well as the entire plArB element may be retained (Figure S4). All of these alternative outcomes were readily identified by PCR (Table 2). Eight out of 18 MS2 loop-containing excisions of *roX1^[MS2-6]T2A^* retained a 3′ P-end. However, one of these is an imprecise excision that is mutated for *w^+mC^* but retains both *P*-elements in tandem (Figure 3D). In agreement with the structure determined by PCR, this line also produced both 547-bp and 869-bp PCR amplicons when tested for presence of MS2 loops in *roX1*. Two out of 12 excisions of *roX1^[MS2-6]T4B^* retained the 3′ P-end and plArB (Figure S4). No residual *P*-element sequences were detected in the eight excisions of *roX1^[MS2-6]R36A^* examined (Table 2). We conclude that the overwhelming majority of excisions are repaired by a mechanism consistent with template-directed gap repair. Sixty-one percent of these had eliminated all vector sequences.

**Table 2:** Retention of P-element sequences

Parent Line	Flies with MS2 Loops	3′ P-End	P-Element Junction
*roX1^[MS2-6]T2A^*	18	8	1*^a^*
*roX1^[MS2-6]T4B^*	12	2*^b^*	2*^b^*
*roX1^[MS2-6]R36A^*	8	0	0

Parent Line	Flies Without MS2 Loops	3′ P-End	P-Element Junction
*roX1^[MS2-6]T2A^*	13 (out of 150)	12	0
*roX1^[MS2-6]T4B^*	10 (out of 90)	6	0
*roX1^[MS2-6]R36A^*	12 (out of 71)	0	0

a Imprecise excision.

b Two rearrangements retained plArB and the 3′ P-end of p[*w^+mC^* GM *roX1^MS2-6^*].

To confirm the structure of rearranged chromosomes, representative lines were analyzed by DNA blotting using the *roX1* promoter region as probe (Figure 3F). Excisions 2A.1 and 4B.1 are conversions to *roX1^MS2-6^* that retain no P-ends. Each produces a single 5.2-kb hybridizing *Eco*R1 fragment, consistent with introduction of 322 bp MS2 loops into the 4.9-kb genomic *Eco*R1 fragment. Line 36A.1, which displayed contraction of the MS2 loop array, shows a single hybridizing band at 5.1 kb (Figure 3 E and F). Line 2.1 retains no *P*-element sequences and has repaired *roX1* without incorporating MS2 loops. As expected, a single 4.9-kb band is detected in this line. In contrast, the imprecise excision line 2.5 described has two hybridizing bands. The *Eco*R1 fragment present in p[*w^+mC^* GM *roX1^MS2-6^*] is 5.2 kb, and a 2.5-kb band, consistent with insertional disruption of the chromosomal *roX1* gene, is also present.

### The *roX1^MS2-6^* is functional in dosage compensation

The *roX1* is functionally redundant with *roX2* for dosage compensation. We tested the engineered *roX1^MS2-6^* allele for *roX* activity by determining adult male survival after recombination with *roX2Δ*, a deletion of *roX2* (Menon and Meller 2012). Male flies inheriting *roX1^MS2-6^ roX2Δ* chromosomes derived from three independent gene conversions were fully viable (Table 3).

**Table 3:** *roX1^MS2-6^* retains *roX1* activity

*roX1^MS2-6^* Line	Mother	Father	Daughters	Sons
2A.1	*roX1^MS2-6^ roX2Δ*	++/Y	100% (1048)	96% (1001)
4B.1	*roX1^MS2-6^ roX2Δ*	++/Y	100% (480)	99% (474)
36A.1	*roX1^MS2-6^ roX2Δ*	++/Y	100% (661)	99% (654)

Male survival is based on the number of females emerging from each mating. Total numbers of flies recovered are provided in parentheses.

### Mobilization of targeted insertions in females

Although *roX1^MS2-6^* was produced with high efficiency, excision was performed in males. Because *roX1* is X-linked, no alternative template for repair is present. It is possible that mobilization in females would be less efficient because of selection of the homolog, rather than the sister chromatid, as the repair template. To test this idea, we mobilized the tandem insertion *roX1^[MS2-6]T2A^* in females. Only 3 out of 131 white-eyed sons incorporated MS2 loops into the *roX1* locus. This efficiency, 2.3%, contrasts with more than 10% MS2 loop incorporation in the offspring of dysgenic males. Two of the three lines contained a 3′ P-end, and thus represent an alternative rearrangement.

Reduced efficiency of MS2 loop incorporation could result from use of *roX1^+^* on the balancer chromosome as the repair template. Alternatively, it could reflect differences in the repair process in the male and female germ lines. For example, if repair tracts tend to be shorter in females, then inclusion of MS2 loops would be less frequent. To address these possibilities, we searched for *P*-element sequences on the repaired chromosomes. Retention of P-ends is expected when a sister chromatid template is utilized. We examined 125 randomly selected white-eyed offspring (including three with MS2 loops) for the presence of a 3′ end; 103 out of 125 (82.4%) retained the 3′ end. We then selected 29 flies at random (out of 125) and tested for the junction between the 3′ end of p[*w^+^*^mC^ GM *roX1^MS2-6^*] and plArB. Twenty-one (72.4%) retained the junction. These findings are consistent with the idea that template-directed gap repair in females strongly favors copying of the sister chromatid.

### Visualization of *roX1* localization in *roX1^MS2-6^* embryos

**Figure 4:**
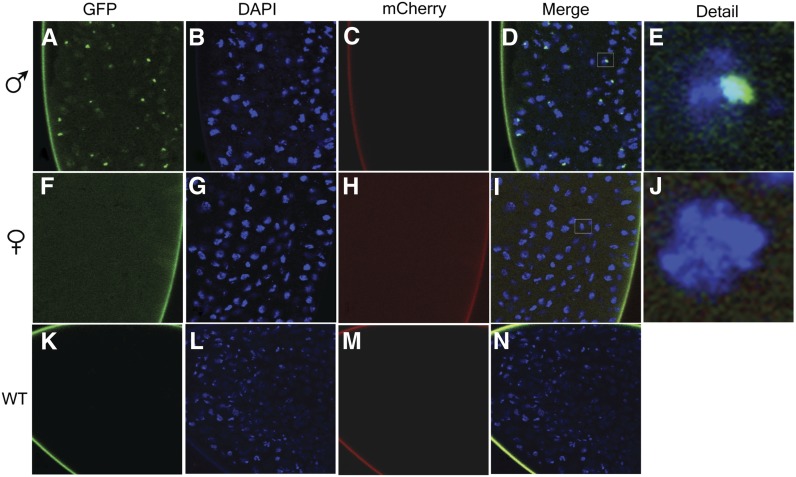
The *roX1^MS2-6^* supports focal recruitment of MCP-GFP in male embryonic nuclei. Embryos were generated by mating *roX1^MS2-6^ roX2Δ*; [*w^+mC^* MCP-GFP] females to males carrying an X-linked [*w^+mC^ Sqh-mCherry*] transgene. Sons (*roX1^MS2-6^ roX2Δ/Y*; [*w^+mC^* MCP-GFP]/+) lack *w^+mC^ Sqh-mCherry* (A–E). Females express *mCherry* (F–J). A wild-type embryo reveals autofluorescence limited to the vitelline membrane (K–N). Details in (E) reveal MCP-GFP recruitment to a single domain within the male nucleus, consistent with X-chromosome painting. MCP-GFP recruitment is absent in the female nucleus (I, J). Each set of panels is derived from a single Z-plane image. The brightness of *mCherry* signals was uniformly enhanced for reproduction (C, H, and M). See *Materials and Methods* for details of photography and image processing.

To visualize *roX1* distribution in embryos, *roX1^MS2-6^ roX2Δ* stocks carrying p[*w^+^*^mC^ MCP-GFP] were generated. Females (*roX1^MS2-6^ roX2Δ*; [*w^+^*^mC^ MCP-GFP]) were mated to males carrying an X-linked p[*w^+^*^mC^ *Sqh-mCherry*] insertion. All embryos from this mating carry the *roX1^MS2-6^ roX2Δ* X chromosome and a single copy of p[*w^+^*^mC^ MCP-GFP], but females display weak *mCherry* expression throughout (Figure 4, compare 4C and 4H). MCP-GFP is recruited to a single, large, subnuclear domain in male (Figure 4, A–E) but not female (Figure 4, F–J) embryos. MCP-GFP in males overlaps the nuclear DAPI signal, and the domain occupied is a size consistent with X-chromosome painting (Figure 4E). Examination of confocal Z-stacks from individual embryos reveals single GFP foci in virtually every nucleus (File S1, avi file).

## Discussion

The *roX* RNAs occupy a central position in fly dosage compensation. Full upregulation of X-linked genes does not occur in male *roX1 roX2* mutants, and the MSL proteins mislocalize to ectopic autosomal sites (Meller and Rattner 2002; Deng and Meller 2006). Although autosomal *roX* transgenes rescue *roX1 roX2* males, these transgenes also recruit MSL proteins to flanking autosomal chromatin, which is then modified in a manner similar to that at compensated X-linked genes (Kelley *et al.* 1999; Henry *et al.* 2001; Kelley and Kuroda 2003; Oh *et al.* 2004; Larschan *et al.* 2007). These observations suggest that position of *roX* genes on the X chromosome contributes to their normal function. More generally, the presence of complex or distant regulatory elements, or a requirement for a specific chromatin context, may contribute to deficiencies in the function of transgenics. Our objective was to generate an allele of *roX1* that would function normally yet be readily visualized by GFP. The engineered allele *roX1^MS2-6^* supports full male viability in a *roX2Δ* background. Visualization of *roX1^MS2-6^* RNA with MCP-GFP reveals punctate labeling of a subnuclear domain in male embryos and does not require lengthy histological protocols, making *roX1^MS2-6^* a new resource for detection of *roX1* localization.

The absence of readily accomplished homologous recombination in *Drosophila* is a notable drawback in a powerful model organism. Groundbreaking studies more than a decade ago established a technique for homologous recombination in flies, but this process remains labor-intensive (Rong and Golic 2000; Gao *et al.* 2008; Huang *et al.* 2009; Wesolowska and Rong 2010). More recently, a strategy for reinsertion of large clones that has been modified by recombineering has been shown to be quite efficient (Bateman *et al.* 2013). This and similar strategies that use site-specific recombination leave vector remnants or recombination sites within the genome (Crown and Sekelsky 2013). In contrast, we have introduced an engineered change with no residual vector sequences. Alternative rearrangements that retain a P-end can be predicted and easily detected by PCR.

We have named this new strategy Targeted Gene Conversion (TGC) to reflect the two-step process required: targeted transposition followed by gene conversion. TGC is a variation of older techniques that utilized repair-mediated gene conversion to engineer *Drosophila* genes. These relied on transposon mobilization to generate double-stranded breaks that were then repaired using a template supplied by the homolog (Gloor *et al.* 1991; Johnson-Schlitz and Engels 1993), by a transposon at another position in the genome (Nassif *et al.* 1994; Lankenau *et al.* 1996; Merli *et al.* 1996), or by DNA injected into dysgenic embryos (Banga and Boyd 1992). The efficiency of this process, typically not exceeding a few percent of excised chromosomes, has limited its use. In contrast, almost all excisions of targeted insertions containing the template are repaired using the template, and 10% of these incorporated MS2-6 loops into *roX1*.

Directed mutagenesis has been improved by the use of zinc finger nucleases (ZFN) and, more recently, TALENs and CRISPR/Cas9 nucleases, to introduce double-stranded breaks at specific sites (Bibikova *et al.* 2002; Christian *et al.* 2010; Bassett *et al.* 2013; Gratz *et al.* 2013). When repair templates with the desired changes are present, these sequences may be introduced by gene conversion (Gaj *et al.* 2013). The ability to rapidly generate mutations at a specific site is a clear strength of these methods. However, the efficiency of gene conversion decreases sharply with an increased distance from the break site (Moehle *et al.* 2007).

The potential for efficient replacement of longer sequences is anticipated to be a primary strength of TGC. Generation of *roX1^SMC17A^* required resection of more than 1.2 kb from the break site, followed by copying more than 3 kb of sequence, including the entire LacZ gene, into the break. Because this precise rearrangement accounted for 68% of excisions, TGC readily replaced large blocks of sequence. Gene conversion was also easily achieved on mobilization of *roX1^[MS2-6]T^* or *roX1^[MS2-6]R^* but, in this instance, no resection of broken ends was necessary to uncover homology with p[*w^+mC^* GM *roX1^MS2-6^*]. Instead, incorporation of MS2 loops requires a repair tract to extend at least 750 bp from the break and to accommodate 322 bp of nonhomologous sequence. Ten percent of excisions incorporate MS2 loops, consistent with a previous study that documented conversion tracts extending almost 2 kb (Nassif and Engels 1993).

The *roX1^Δ891^* and three targeted *roX1^[MS2-6]^* insertions are readily mobilized by transposase, with more than 90% of dysgenic males producing white-eyed offspring. This is not a general feature of *P*-element insertions in *roX1*, because only 20% of dysgenic *roX1^mb710^* males produce *ry* offspring (V. H. Meller, unpublished results). Despite high mobility, recovery of imprecise excisions was remarkably low. Four out of 56 excisions of *roX1^Δ891^* and 4 out of 352 excisions of targeted *roX1^[MS2-6]^* insertions were imprecise. The apparent high mobility and bias against imprecise excision are likely attributable to the presence of an alternative template for gap repair that excludes *w^+mC^*.

A clear limitation of our strategy is the need to move the template sequence close to the target site. We have accomplished this by targeted transposition, but targeted transpositions typically comprise a few percent of new insertions and require a *P*-element at the target site. The exceptionally rich coverage of *P*-element insertions in *Drosophila* makes this feasible in many instances. Alternatively, recently developed techniques that use engineered nucleases, such as TALENs, CRISPR/Cas9, or ZFNs, could be used to introduce a landing site, such as *attP*, at the desired location (Groth *et al.* 2004; Gaj *et al.* 2013). Integration of a selectable marker and template flanked by P-ends would generate a mutagenic precursor for TGC without the need for a preexisting *P*-element (Figure S6).

## Supplementary Material

Supporting Information
