# Supplementary material for: Generation of a Useful roX1 Allele by Targeted Gene Conversion
Source: G3 (Bethesda). 2013 Nov 26;4(1):155–62. doi: 10.1534/g3.113.008508 (PMC3887531; doi:10.1534/g3.113.008508)
Supplement: Supporting Information [file supp_4_1_155__index.html]

Generation of a Useful roX1 Allele by Targeted Gene Conversion — Supporting Information 

# Generation of a Useful *roX1* Allele by Targeted Gene Conversion

## Supporting Information for Apte *et al.*, 2014

**Files in this Data Supplement:**

- Supporting Information - Figures S1-S6, File S1, and Table S1 (PDF, 1 MB)
- Figure S1 - Overview of *roX1SMC17A* creation. (PDF, 626 KB)
- Figure S2 - Rearrangements produced by *roX1Δ891* mobilization. (PDF, 620 KB)
- Figure S3 - Proposed mechanism capable of producing class 4 rearrangements. (PDF, 531 KB)
- Figure S4 - Products of gap repair generated by mobilization of tandem insertion *roX1[MS2-6]T4B*. (PDF, 652 KB)
- Figure S5 - Predicted products of gap repair upon mobilization of tandem insertion *roX1[MS2-6]R36A*. (PDF, 634 KB)
- Figure S6 - Directing transposase-mediated gene conversion to a region lacking a P-element. (PDF, 494 KB)
- Table S1 - Primer sequences used for characterization of *roX1* rearrangements (5'- 3'). (PDF, 554 KB)
- File S1 - MCP-GFP recruitment to a single domain within the male nucleus (.zip, 629 KB)
