# Supplementary material for: Generation of a Useful roX1 Allele by Targeted Gene Conversion
Source: G3 (Bethesda). 2013 Nov 26;4(1):155–62. doi: 10.1534/g3.113.008508 (PMC3887531; doi:10.1534/g3.113.008508)
Supplement: Supporting Information [file supp_g3.113.008508_008508SI.pdf]

G3: Genes | Genomes | Genetics

Generation of a useful *roX1* allele by Targeted Gene Conversion

Manasi S. Apte*, Victoria A. Moran*, Debashish U. Menon*, Barbara P. Rattner[§,2], Kathryn Hughes Barry[§,3], Rachel M. Zunder[§], Richard Kelley[†] and Victoria H. Meller*[1].

* Department of Biological Sciences, Wayne State University, MI, USA, 48202
[§] Department of Biology, Tufts University, MA, USA, 02155
[†] Department of Molecular and Human Genetics, Baylor College of Medicine, TX, USA, 77030
2 Current address: Landes Biosciences, 1806 Rio Grande St., Austin, TX 78701

3 Current address: Occupational and Environmental Epidemiology Branch, Division of Cancer Epidemiology and Genetics, National Cancer Institute, Rockville, MD, 20892

[1]Corresponding Author:
Victoria H. Meller,
Dept. of Biological sciences,
Wayne state university, Detroit, MI, 48202
Phone: 313-577-3451
Fax: 313-577-6891
vmeller@biology.biosci.wayne.edu

DOI: 10.1534/g3.113.008508

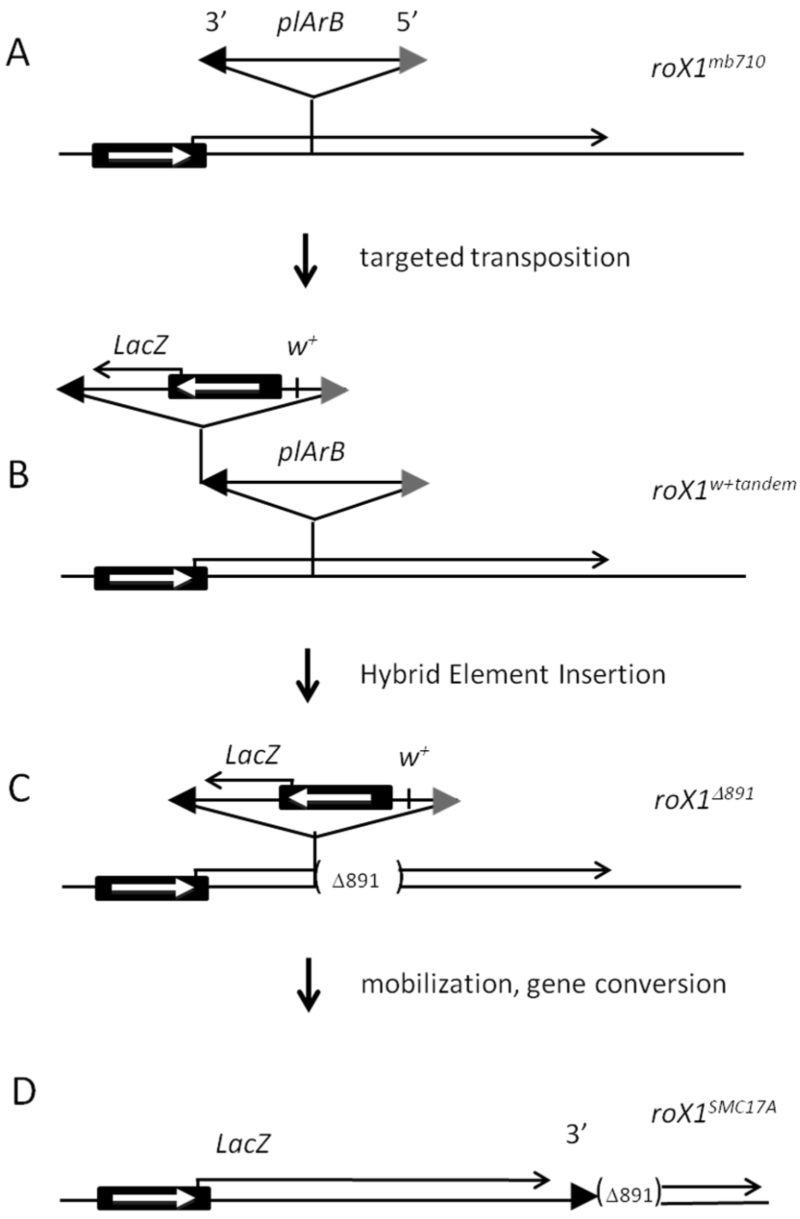

Figure S1 Overview of *roX1^{SMC17A}* creation. A) *roX1^{mb710}* is created by insertion of plArB. **B)** p[*w^{+mC} roX1P*-βgal], containing the *roX1* promoter (white arrow) fused to LacZ, was moved into *roX1* by targeted transposition. The resulting tandem insertion (*roX1$^{w+tandem}$*) was the starting point for Hybrid Element Insertion mutagenesis that removed plArB and deleted 891 bp flanking the insertion site, producing *roX1$^{\Delta 891}$* (**C**). Mobilization of p[*w^{+mC} roX1P*-βgal] produced *roX1^{SMC17A}* (**D**), and numerous identical rearrangements. The *roX1^{SMC17A}* chromosome carries the fusion of *LacZ* with the *roX1* promoter that is present in p[*w^{+mC} roX1P*-βgal]. All *roX1* sequences between the promoter and the 5' P-end have been replaced with a full length LacZ gene. The 5' P-end has been replaced precisely with the 3' end. A complete list of the rearrangement classes produced by *roX1$^{\Delta 891}$* mobilization, and a model for the homology-dependent gene conversion event that likely produced *roX1^{SMC17A}*, is presented in Supplementary Figure 2.

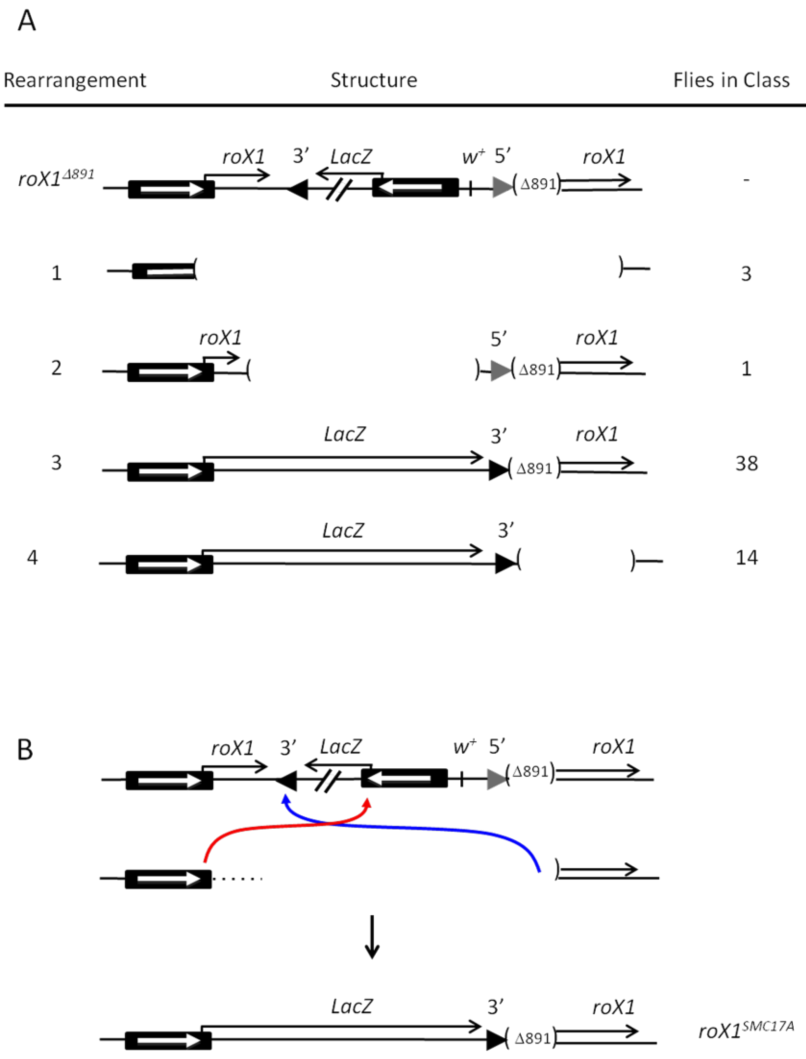

Figure S2 Rearrangements produced by *roX1$^{\Delta891}$* mobilization. **A)** Four classes of rearrangements were present in white eyed offspring of dysgenic *roX1$^{\Delta891}$* flies (top). The *roX1* promoter is depicted by a white arrow. Imprecise excisions that remove all (class 1) or the 3' end (class 2) of p[*w^{+mC} roX1P*-βgal] occurred in 4 flies. Rearrangements identical to *roX1^{SMC17A}* (class 3) were recovered 38 times. Rearrangements similar to *roX1^{SMC17A}*, but with the 3' P-end missing, or inserted at a different location, account for 14 flies (class 4). A hypothetical mechanism for generating class 4 is presented in Supplemental Figure 3. **B)** Excision followed by resection reveals homology between the *roX1* promoters on the chromosome and in p[*w^{+mC} roX1P*-βgal] (red arrow). Homology is also present at the 3' P-end on the sister chromatid and at the site where the 5' P-end excised (blue arrow). We postulate that these homologies support gap repair using a sister chromatid template. This will insert the full length *LacZ* gene into *roX1* and substitute the 3' P-end for the original 5' end, the precise rearrangement found in *roX1^{SMC17A}* (bottom). Drawings not to scale.

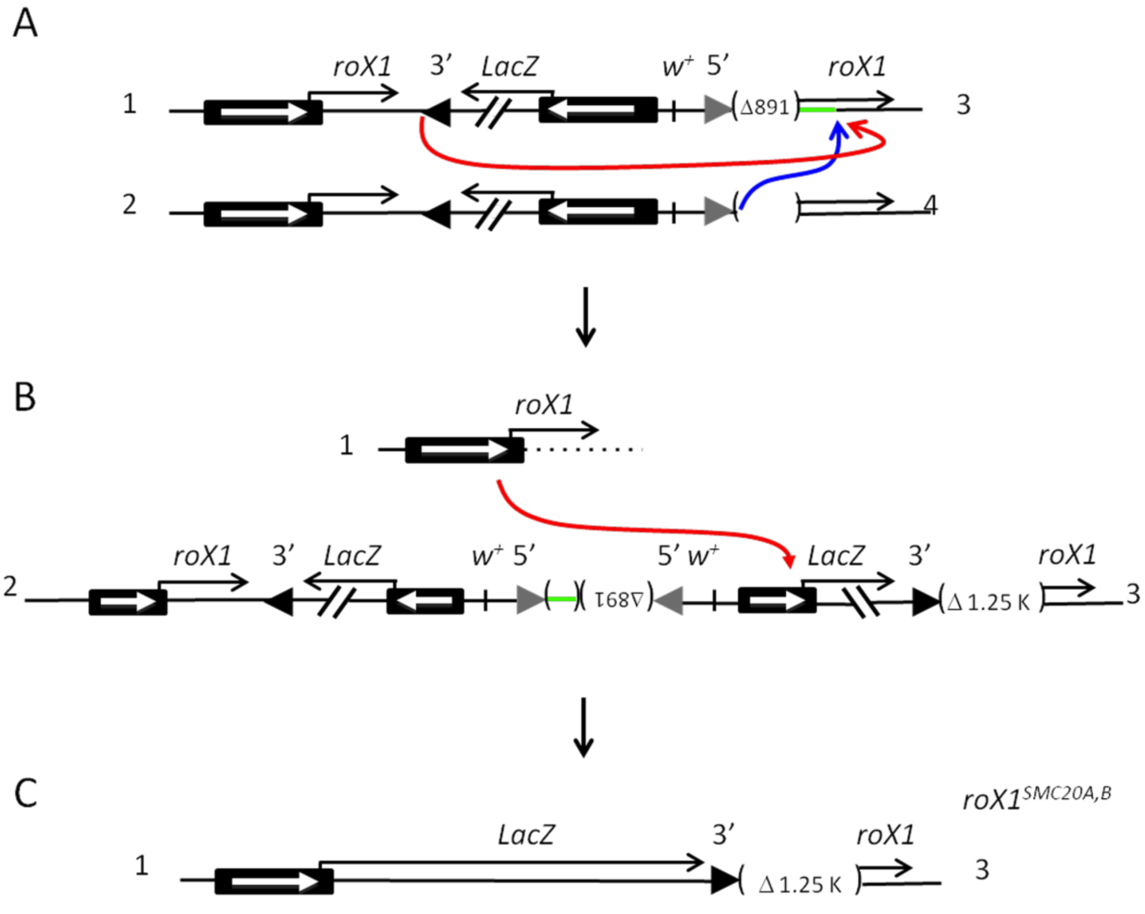

Figure S3 Proposed mechanism capable of producing class 4 rearrangements. **A)** Hybrid Element Insertion (HEI) creates an inverted duplication of p[w^{+mC} *roX1P*-βgal] on one chromatid, depicted in B. Red and blue arrows show the insertion sites of the 3’ and 5’ P-ends participating in HEI. Proximal (1,2) and distal (3,4) chromatid ends are labeled. HEI places a 3’ P-end downstream from the 5’ end in $roX1^{\Delta 891}$. A green genomic fragment from *roX1* now appears in inverted orientation between the p[w^{+mC} *roX1P*-βgal] elements in B. **B)** Chromatid arm 1 is resected to reveal homology to the *roX1* promoter. Broken arm 1 initiates recombinational repair with the *roX1* promoter in p[w^{+mC} *roX1P*-βgal] (red arrow). **C)** Resolution produces a chromosome carrying the *roX1* promoter fused to LacZ. The 5’ P-end has been replaced by a 3’ P-end that is downstream from the insertion sites in $roX1^{\Delta 891}$ and $roX1^{SMC17A}$. This model is consistent with the structure of $roX1^{SMC20A,B}$, identical to $roX1^{SMC17A}$ but with the 3’ P-end moved 350 bp, creating a deletion of 1.25 kb. Twelve additional flies in this class also had the *roX1* promoter fused to LacZ, but no P-end could be detected using primers in *roX1*. We postulate that these rearrangements were similarly produced, but that the HEI insertion occurred distal to *roX1*.

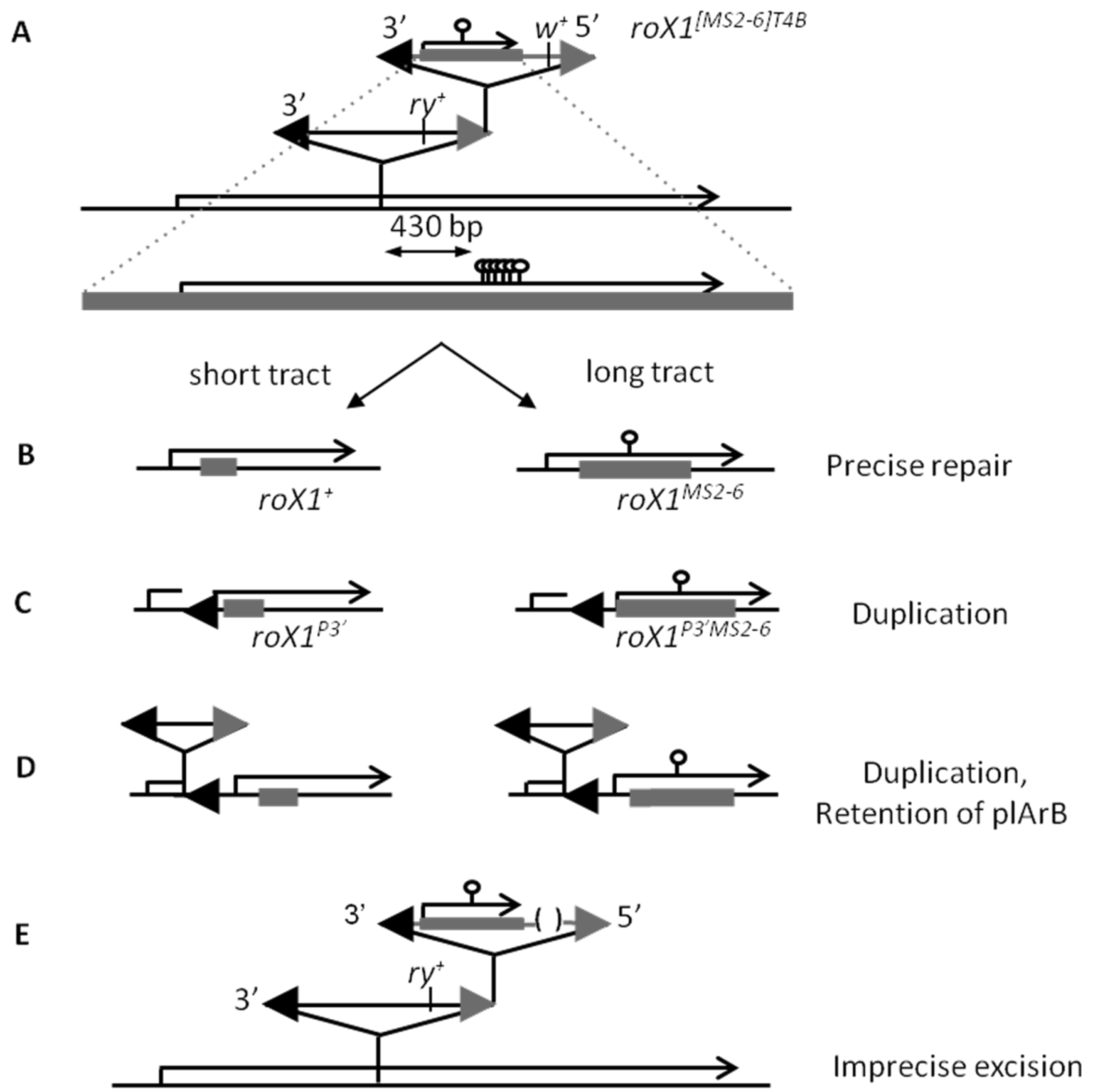

Figure S4 Products of gap repair generated by mobilization of tandem insertion *roX1*$^{[MS2\text{-}6]T4B}$. A) *roX1*$^{[MS2\text{-}6]T4B}$. The *roX1*$^{MS2\text{-}6}$ insert (heavy gray line) is shown collinear to and below he corresponding genomic sequence. The MS2 loops are 430 bp from the plArB insertion site in *roX1*. Predicted products of homology-dependent gap repair presented in (B-D). Left panels depict short repair tracts (gray) that do not incorporate MS2 loops, right panels are longer tracts incorporating MS2 loops into the chromosome. **B)** Precise replacement by *roX1*$^{MS2\text{-}6}$ sequences. **C)** Repair is supported by homology in *roX1* and at the 3' P-end, leading to retention of a P-end and duplication of the 5' *roX1*. **D)** Retention of plArB. **E)** Imprecise excision mutates mini-white, but leaves both P-elements in place.

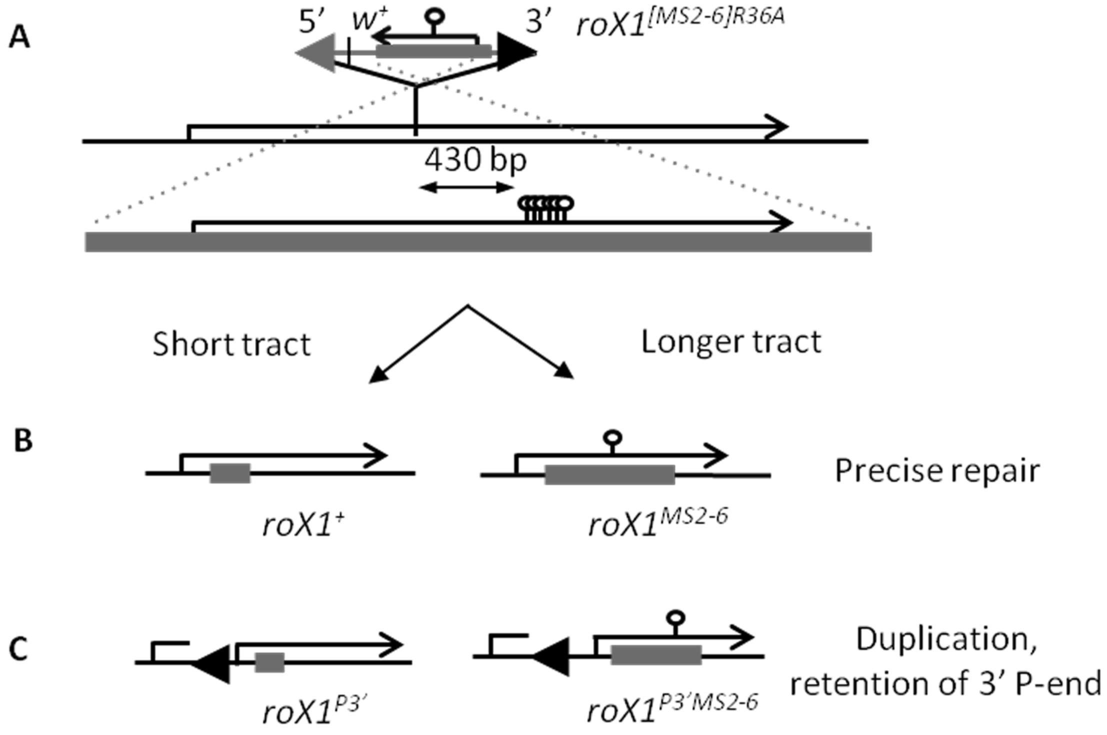

Figure S5 Predicted products of gap repair upon mobilization of tandem insertion *roX1$^{[MS2-6]R36A}$*. A) *roX1$^{[MS2-6]R36A}$* has replaced plArB with p[w^{+mC} GM *roX1^{MS2-6}*]. *roX1^{MS2-6}* (heavy gray line) is shown collinear to and below he corresponding genomic sequence. The MS2 loops are 430 bp from the plArB insertion site in the *roX1*. Predicted products of homology dependent gap repair are depicted in B (precise repair of *roX1*) and C (retention of the 3' P-end and duplication of 5' *roX1* sequences). Left panels depict short repair tracts that do not incorporate MS2 loops, right panels describe longer tracts incorporating MS2 loops into the chromosome.

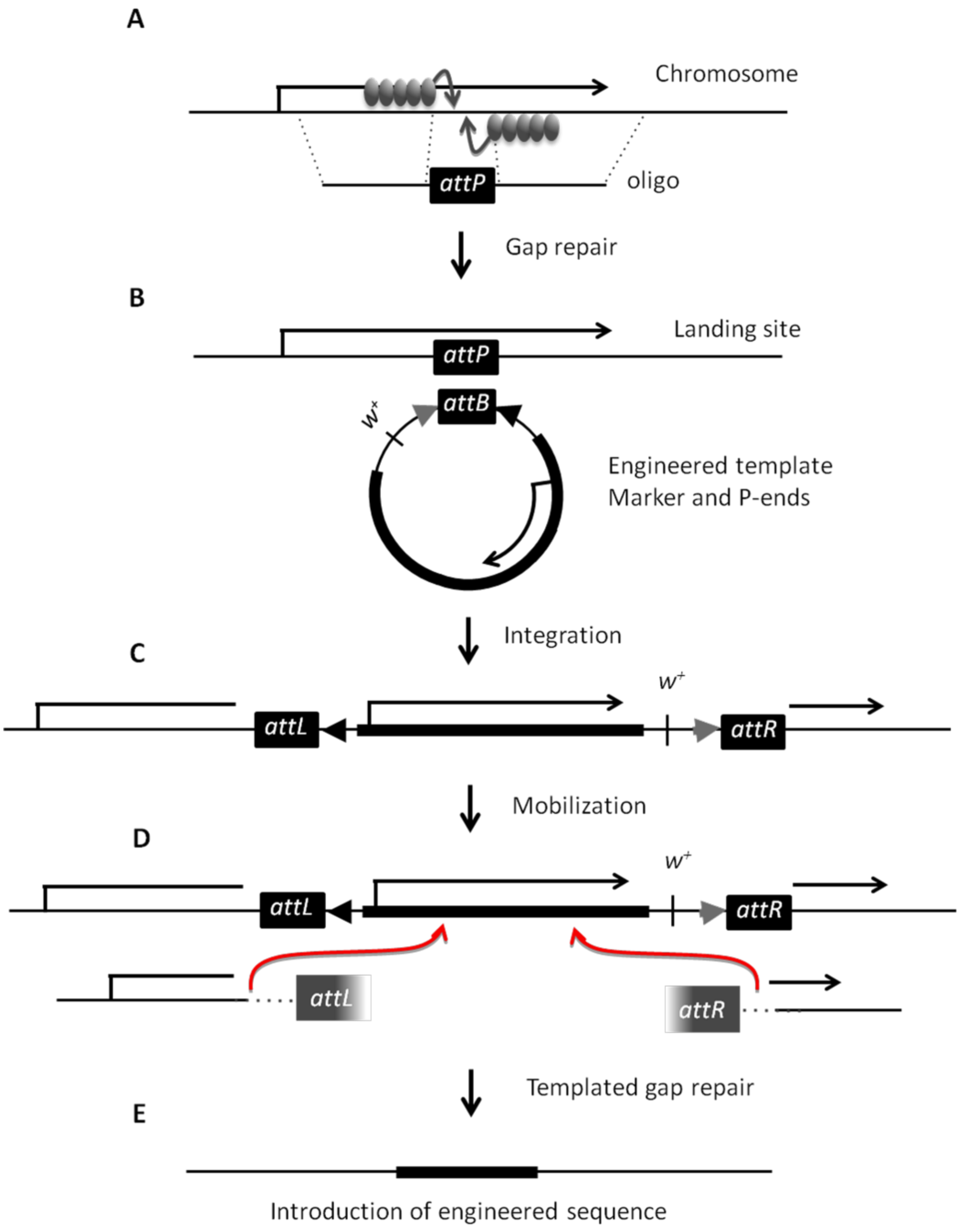

Figure S6 Directing transposase-mediated gene conversion to a region lacking a P-element. A) A double stranded break is introduced in a gene of interest by an engineered nuclease. An oligonucleotide containing a landing site, such as *attP*, and homologous arms is introduced as a repair template. **B)** A longer construct with engineered changes to the target gene (thick line), a visible marker (*w*+) and P-ends (black and gray arrowheads) is integrated into the landing site **(C). D)** Mobilization with transposase creates a double stranded break. Homology is revealed by resection of broken ends. Gap repair using a sister chromatid template produces engineered chromosomes lacking the *w*+ marker.

File S1

MCP-GFP recruitment to a single domain within the male nucleus

File S1 A and B are available for download as .avi files at

http://www.g3journal.org/lookup/suppl/doi:10.1534/g3.113.008508/-/DC1

Table S1 Primer sequences used for characterization of *roX1* rearrangments (5'- 3')

Pry2	CTTGCCGACGGGACCACCTTATGTTATT
Pry4(+)	TAATCAACAATCATATCGCTGTCTCACTCAG
pLac1(+)	CCAAGGCTGCACCCAAGGCTCTGCTCCCAC
BPR10	GAGGACCCGGGTAGAGCGCATAGCTCTTG
BPR15	CGGAACGAAAGAGACAAATG
$roX1^{ex6}F$	GCTCTAGAATTCGAAAGTTGCGTATAACGG
BPR19	GATGGCCTTCAGTTGGTG
