# Supplementary material for: Generation of a Useful roX1 Allele by Targeted Gene Conversion
Source: G3 (Bethesda). 2013 Nov 26;4(1):155–62. doi: 10.1534/g3.113.008508 (PMC3887531; doi:10.1534/g3.113.008508)
Supplement: Supporting Information [file supp_g3.113.008508_FigureS1.pdf]

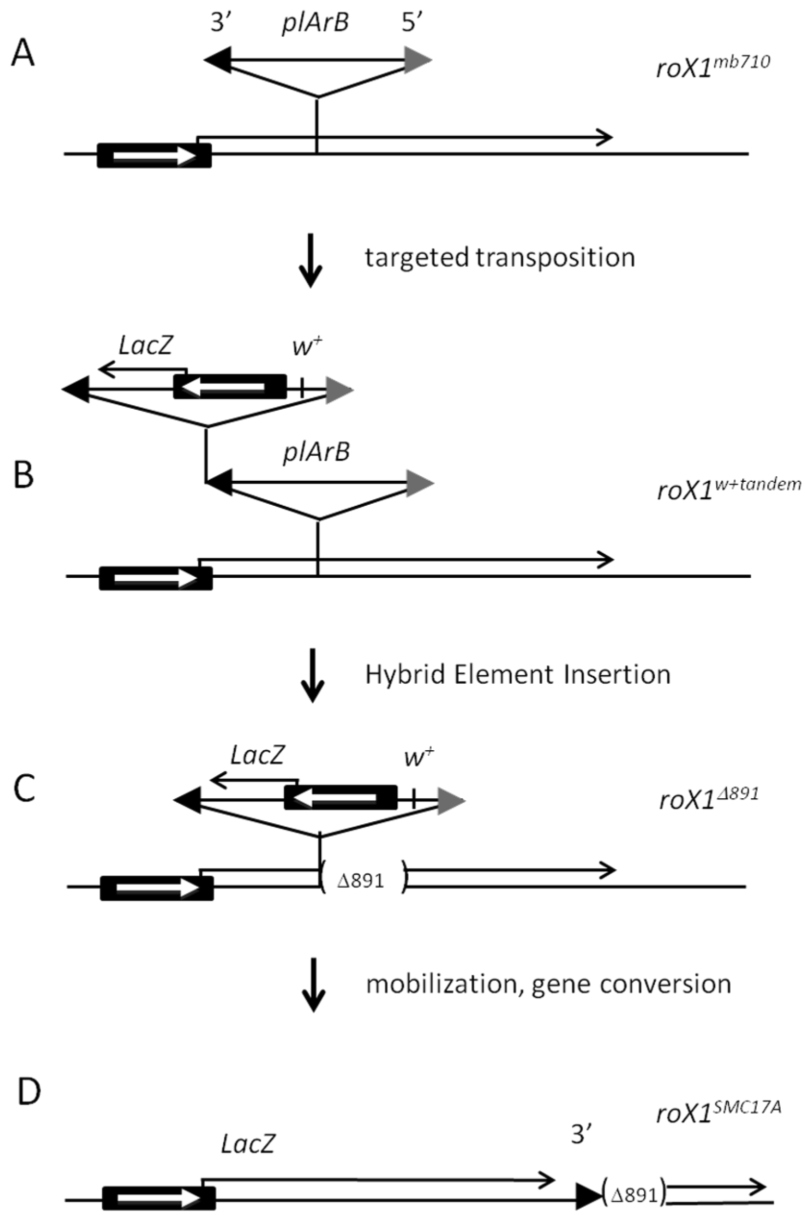

Figure S1 Overview of *roX1*^{SMC17A} creation. A) *roX1*^{mb710} is created by insertion of plArB. **B)** p[w^{+mC} *roX1P*-βgal], containing the *roX1* promoter (white arrow) fused to LacZ, was moved into *roX1* by targeted transposition. The resulting tandem insertion (*roX1*$^{w+tandem}$) was the starting point for Hybrid Element Insertion mutagenesis that removed plArB and deleted 891 bp flanking the insertion site, producing *roX1*$^{\Delta 891}$ (**C**). Mobilization of p[w^{+mC} *roX1P*-βgal] produced *roX1*^{SMC17A} (**D**), and numerous identical rearrangements. The *roX1*^{SMC17A} chromosome carries the fusion of *LacZ* with the *roX1* promoter that is present in p[w^{+mC} *roX1P*-βgal]. All *roX1* sequences between the promoter and the 5' P-end have been replaced with a full length LacZ gene. The 5' P-end has been replaced precisely with the 3' end. A complete list of the rearrangement classes produced by *roX1*$^{\Delta 891}$ mobilization, and a model for the homology-dependent gene conversion event that likely produced *roX1*^{SMC17A}, is presented in Supplementary Figure 2.
