# Supplementary material for: Generation of a Useful roX1 Allele by Targeted Gene Conversion
Source: G3 (Bethesda). 2013 Nov 26;4(1):155–62. doi: 10.1534/g3.113.008508 (PMC3887531; doi:10.1534/g3.113.008508)
Supplement: Supporting Information [file supp_g3.113.008508_FigureS2.pdf]

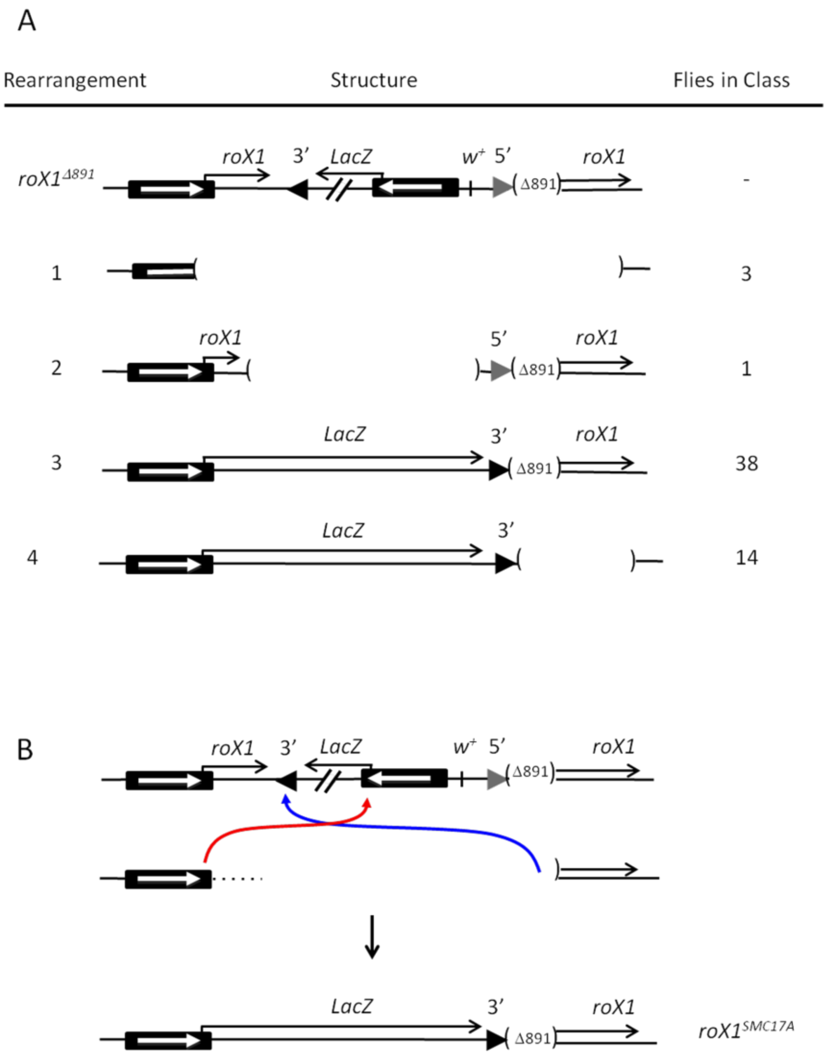

Figure S2 Rearrangements produced by *roX1*$^{\Delta 891}$ mobilization. **A)** Four classes of rearrangements were present in white eyed offspring of dysgenic *roX1*$^{\Delta 891}$ flies (top). The *roX1* promoter is depicted by a white arrow. Imprecise excisions that remove all (class 1) or the 3' end (class 2) of p[w^{+mC} *roX1P*-βgal] occurred in 4 flies. Rearrangements identical to *roX1*^{SMC17A} (class 3) were recovered 38 times. Rearrangements similar to *roX1*^{SMC17A}, but with the 3' P-end missing, or inserted at a different location, account for 14 flies (class 4). A hypothetical mechanism for generating class 4 is presented in Supplemental Figure 3. **B)** Excision followed by resection reveals homology between the *roX1* promoters on the chromosome and in p[w^{+mC} *roX1P*-βgal] (red arrow). Homology is also present at the 3' P-end on the sister chromatid and at the site where the 5' P-end excised (blue arrow). We postulate that these homologies support gap repair using a sister chromatid template. This will insert the full length *LacZ* gene into *roX1* and substitute the 3' P-end for the original 5' end, the precise rearrangement found in *roX1*^{SMC17A} (bottom). Drawings not to scale.
