# Supplementary material for: Generation of a Useful roX1 Allele by Targeted Gene Conversion
Source: G3 (Bethesda). 2013 Nov 26;4(1):155–62. doi: 10.1534/g3.113.008508 (PMC3887531; doi:10.1534/g3.113.008508)
Supplement: Supporting Information [file supp_g3.113.008508_FigureS3.pdf]

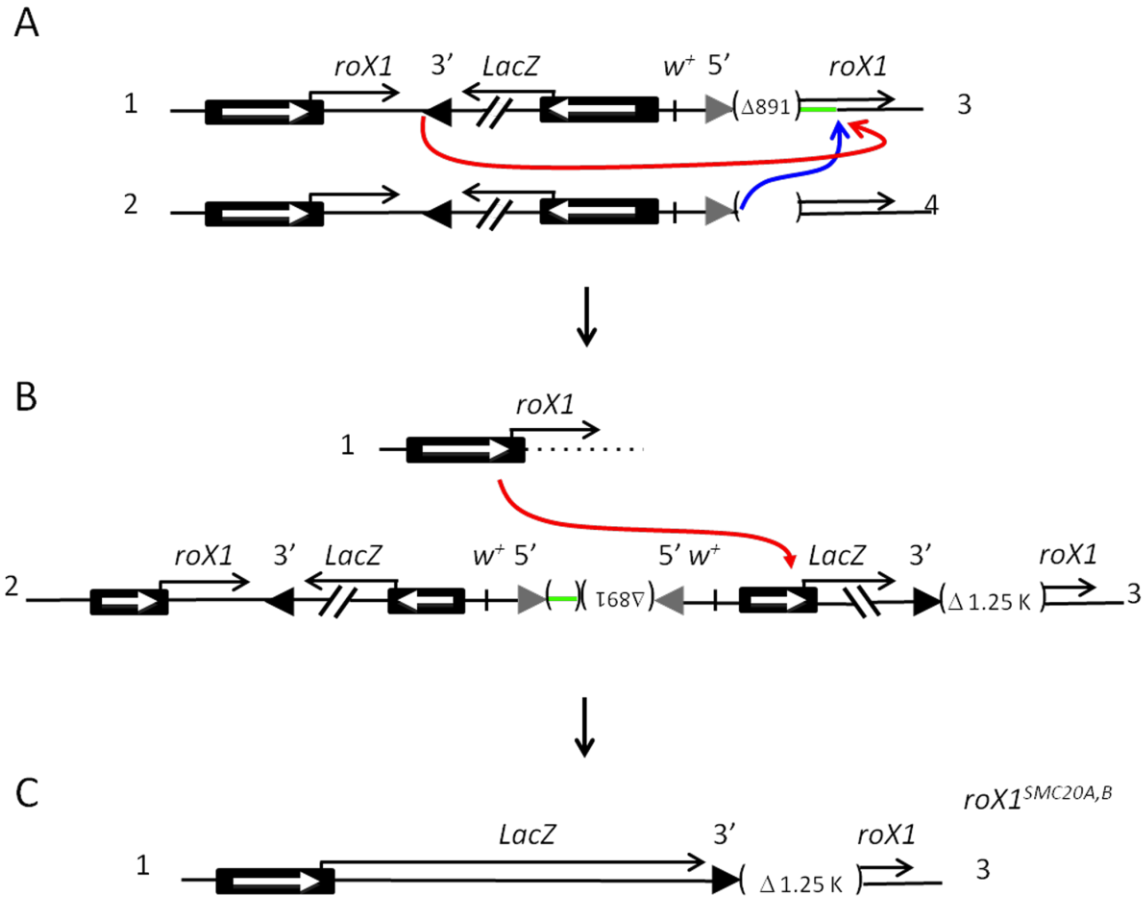

Figure S3 Proposed mechanism capable of producing class 4 rearrangements. **A)** Hybrid Element Insertion (HEI) creates an inverted duplication of p[w^{+mC} *roX1P*-βgal] on one chromatid, depicted in B. Red and blue arrows show the insertion sites of the 3' and 5' P-ends participating in HEI. Proximal (1,2) and distal (3,4) chromatid ends are labeled. HEI places a 3' P-end downstream from the 5' end in $roX1^{\Delta 891}$. A green genomic fragment from *roX1* now appears in inverted orientation between the p[w^{+mC} *roX1P*-βgal] elements in B. **B)** Chromatid arm 1 is resected to reveal homology to the *roX1* promoter. Broken arm 1 initiates recombinational repair with the *roX1* promoter in p[w^{+mC} *roX1P*-βgal] (red arrow). **C)** Resolution produces a chromosome carrying the *roX1* promoter fused to LacZ. The 5' P-end has been replaced by a 3' P-end that is downstream from the insertion sites in $roX1^{\Delta 891}$ and $roX1^{SMC17A}$. This model is consistent with the structure of $roX1^{SMC20A,B}$, identical to $roX1^{SMC17A}$ but with the 3' P-end moved 350 bp, creating a deletion of 1.25 kb. Twelve additional flies in this class also had the *roX1* promoter fused to LacZ, but no P-end could be detected using primers in *roX1*. We postulate that these rearrangements were similarly produced, but that the HEI insertion occurred distal to *roX1*.
