# Supplementary material for: Generation of a Useful roX1 Allele by Targeted Gene Conversion
Source: G3 (Bethesda). 2013 Nov 26;4(1):155–62. doi: 10.1534/g3.113.008508 (PMC3887531; doi:10.1534/g3.113.008508)
Supplement: Supporting Information [file supp_g3.113.008508_FigureS4.pdf]

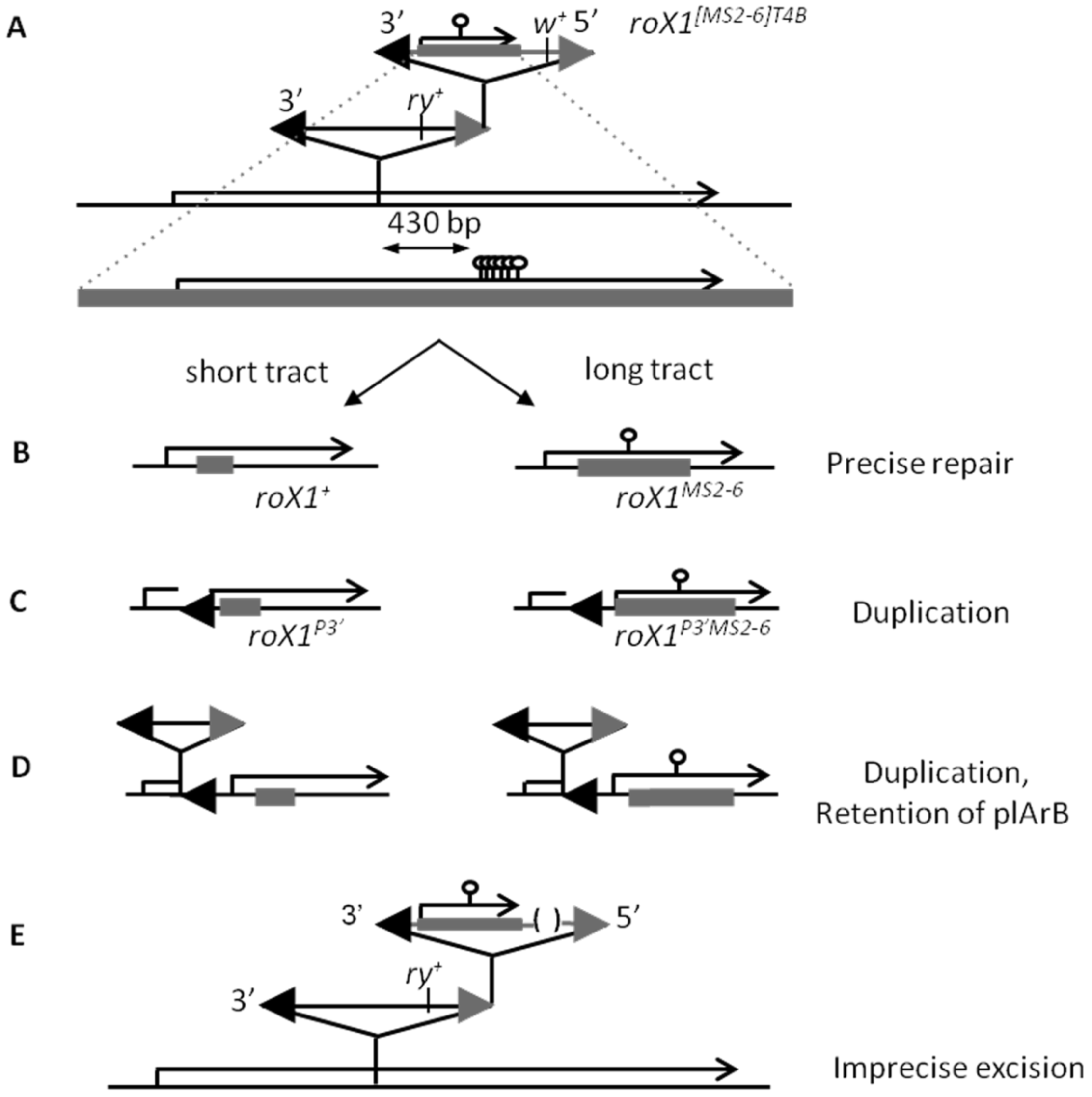

Figure S4 Products of gap repair generated by mobilization of tandem insertion *roX1*$^{[MS2\text{-}6]T4B}$. A) *roX1*$^{[MS2\text{-}6]T4B}$. The *roX1*$^{MS2\text{-}6}$ insert (heavy gray line) is shown collinear to and below he corresponding genomic sequence. The MS2 loops are 430 bp from the plArB insertion site in *roX1*. Predicted products of homology-dependent gap repair presented in (B-D). Left panels depict short repair tracts (gray) that do not incorporate MS2 loops, right panels are longer tracts incorporating MS2 loops into the chromosome. **B)** Precise replacement by *roX1*$^{MS2\text{-}6}$ sequences. **C)** Repair is supported by homology in *roX1* and at the 3' P-end, leading to retention of a P-end and duplication of the 5' *roX1*. **D)** Retention of plArB. **E)** Imprecise excision mutates mini-white, but leaves both P-elements in place.
