# Supplementary material for: Generation of a Useful roX1 Allele by Targeted Gene Conversion
Source: G3 (Bethesda). 2013 Nov 26;4(1):155–62. doi: 10.1534/g3.113.008508 (PMC3887531; doi:10.1534/g3.113.008508)
Supplement: Supporting Information [file supp_g3.113.008508_FigureS5.pdf]

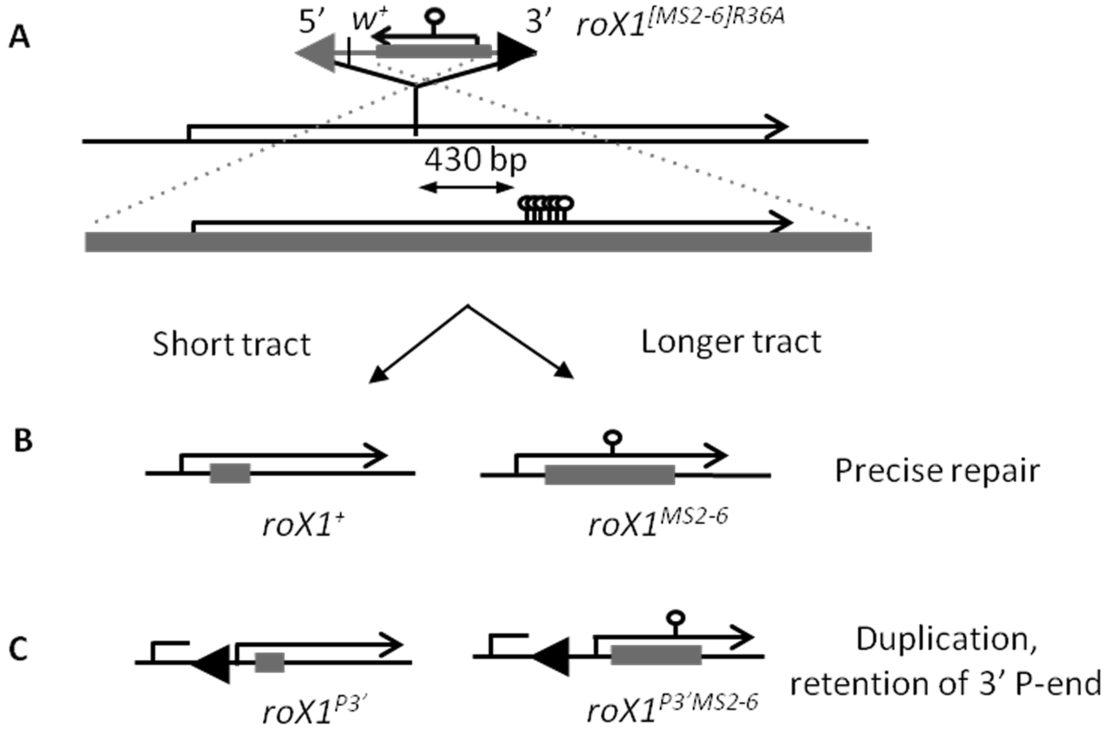

Figure S5 Predicted products of gap repair upon mobilization of tandem insertion *roX1*$^{[MS2\text{-}6]R36A}$. A) *roX1*$^{[MS2\text{-}6]R36A}$ has replaced plArB with p[w^{+mC} GM *roX1*$^{MS2\text{-}6}$]. *roX1*$^{MS2\text{-}6}$ (heavy gray line) is shown collinear to and below he corresponding genomic sequence. The MS2 loops are 430 bp from the plArB insertion site in the *roX1*. Predicted products of homology dependent gap repair are depicted in B (precise repair of *roX1*) and C (retention of the 3' P-end and duplication of 5' *roX1* sequences). Left panels depict short repair tracts that do not incorporate MS2 loops, right panels describe longer tracts incorporating MS2 loops into the chromosome.
