# Supplementary material for: Generation of a Useful roX1 Allele by Targeted Gene Conversion
Source: G3 (Bethesda). 2013 Nov 26;4(1):155–62. doi: 10.1534/g3.113.008508 (PMC3887531; doi:10.1534/g3.113.008508)
Supplement: Supporting Information [file supp_g3.113.008508_FigureS6.pdf]

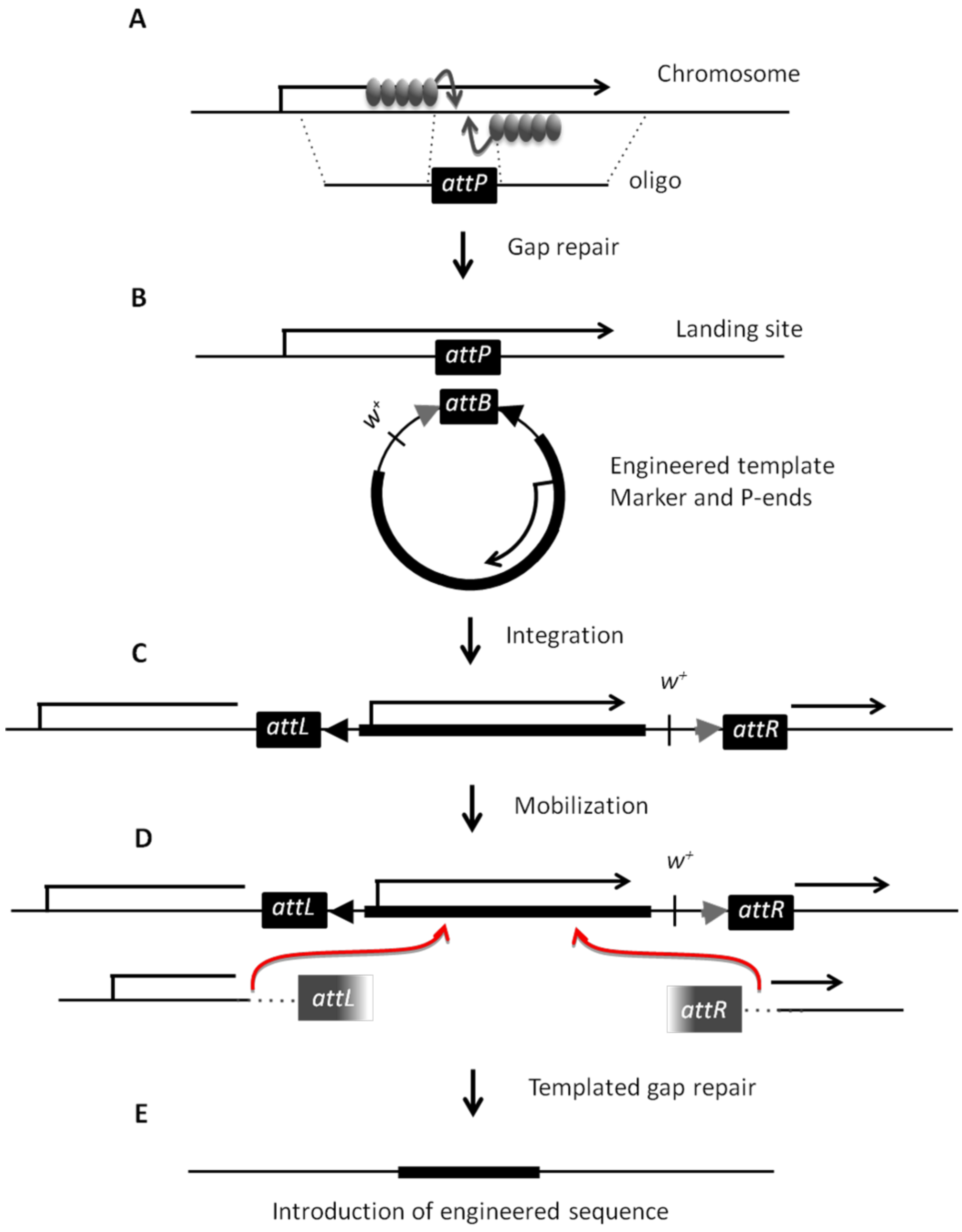

Figure S6 Directing transposase-mediated gene conversion to a region lacking a P-element. A) A double stranded break is introduced in a gene of interest by an engineered nuclease. An oligonucleotide containing a landing site, such as *attP*, and homologous arms is introduced as a repair template. **B)** A longer construct with engineered changes to the target gene (thick line), a visible marker (w^+) and P-ends (black and gray arrowheads) is integrated into the landing site **(C). D)** Mobilization with transposase creates a double stranded break. Homology is revealed by resection of broken ends. Gap repair using a sister chromatid template produces engineered chromosomes lacking the w^+ marker.
