# Supplementary material for: Generation of a Useful roX1 Allele by Targeted Gene Conversion
Source: G3 (Bethesda). 2013 Nov 26;4(1):155–62. doi: 10.1534/g3.113.008508 (PMC3887531; doi:10.1534/g3.113.008508)
Supplement: Supporting Information [file supp_g3.113.008508_TableS1.pdf]

Table S1 Primer sequences used for characterization of *roX1* rearrangments (5'- 3')

Pry2	CTTGCCGACGGGACCACCTTATGTTATT
Pry4(+)	TAATCAACAATCATATCGCTGTCTCACTCAG
pLac1(+)	CCAAGGCTGCACCCAAGGCTCTGCTCCCAC
BPR10	GAGGACCCGGGTAGAGCGCATAGCTCTTG
BPR15	CGGAACGAAAGAGACAAATG
$roX1^{ex6}F$	GCTCTAGAATTCGAAAGTTGCGTATAACGG
BPR19	GATGGCCTTCAGTTGGTG
